# Establishments for Massage or Special Treatment

**Published:** 1916-12-09

**Authors:** James Bird

**Affiliations:** Clerk of the London County Council. London County Council, County Hall, Spring Gardens, S.W.


*To the Editor of* The Hospital.

### Establishments for Massage or Special Treatment.

*To the Editor of* The Hospital.

Sir,—For the guidance of the press and advertising agents dealing with advertisements of establishments for massage or special treatment, the Council desires to state that Orders have been made (i.) refusing registration under Part V. (Establishments for Massage or Special Treatment) of the London County Council (General Powers) Act, 1915, of Madam Marie Meirrschant (Madam Mariette) in respect of the premises No. 131 Jermyn Street, S.W., and (ii.) cancelling the registration of Madam Meirrschant in respect of the establishment at No. 63 Regent Street, S.W.—Yours faithfully,

James Bird,
Clerk of the London County Council.

London County Council, County Hall,
Spring Gardens, S.W., December 1916.

